# Functional Lung Imaging Identifies Peripheral Ventilation Changes in ꞵ‐ENaC Mice

**DOI:** 10.1111/resp.70009

**Published:** 2025-02-25

**Authors:** Nicole Reyne, Ronan Smith, Patricia Cmielewski, Nina Eikelis, Kris Nilsen, Jennie Louise, Julia Duerr, Marcus A. Mall, Mark Lawrence, David Parsons, Martin Donnelley

**Affiliations:** ^1^ Robinson Research Institute, University of Adelaide Adelaide South Australia Australia; ^2^ Adelaide Medical School, University of Adelaide Adelaide South Australia Australia; ^3^ Respiratory and Sleep Medicine, Women's and Children's Hospital North Adelaide South Australia Australia; ^4^ 4DMedical Carlton Victoria Australia; ^5^ Biostatistics Unit, South Australian Health and Medical Research Institute Adelaide Australia; ^6^ Department of Pediatric Respiratory Medicine, Immunology and Critical Care Medicine Charité ‐ Universitätsmedizin Berlin Berlin Germany; ^7^ German Center for Lung Research (DZL), Associated Partner Site Berlin Germany; ^8^ Berlin Institute of Health at Charité‐Universitätsmedizin Berlin Berlin Germany; ^9^ SCIREQ Scientific Respiratory Equipment Inc Montreal Quebec Australia

**Keywords:** animal models, cystic fibrosis, lung disease, lung function, muco‐obstructive, x‐ray velocimetry

## Abstract

**Background and Objective:**

β‐ENaC‐Tg mice serve as a relevant model of muco‐obstructive lung disease and diffuse‐type emphysema, with impaired mucociliary clearance, mucus obstruction, chronic airway inflammation, structural lung damage, and altered lung function. The aim of this study was to undertake a comprehensive analysis of lung function and mechanics of the adult β‐ENaC‐Tg model.

**Methods:**

Adult β‐ENaC‐Tg and wild‐type littermates underwent X‐ray velocimetry (XV) scans using a Permetium XV scanner (4DMedical, Melbourne, Australia). For comparative lung mechanics, lung function assessments were conducted with a flexiVent system (SCIREQ, Montreal, Canada).

**Results:**

XV imaging demonstrated elevated ventilation defect percentage, mean specific ventilation, and ventilation heterogeneity in β‐ENaC‐Tg mice. Spatial analysis of ventilation maps indicated increased ventilation variability in the peripheral lung regions, as well as an increased proportion of under‐ventilated areas. The flexiVent analysis indicated that compared to wild types, β‐ENaC‐Tg mice have a significantly more compliant lungs with increased inspiratory capacity, reduced tissue elastance, and increased hysteresivity (heterogeneity), suggesting loss of parenchymal integrity.

**Conclusion:**

This research highlights the utility of XV imaging in evaluating ventilation defects in the β‐ENaC‐Tg model and provides a comprehensive lung function analysis.

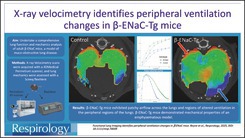

**Summary**

- β‐ENaC‐Tg mice have previously been shown to exhibit muco‐obstructive disease and emphysema.
- This study analysed lung function in β‐ENaC‐Tg mice using XV functional imaging and flexiVent assessments.
- XV enabled ventilation defects to be identified and localised, and the flexiVent comfirmed increased lung compliance and reduced tissue elastance.

## 1 Introduction

A group of muco‐obstructive lung disorders, including cystic fibrosis (CF), chronic obstructive pulmonary disease (COPD), primary ciliary dyskinesia, and non‐cystic fibrosis bronchiectasis, are characterised by reduced mucociliary clearance and increased mucus secretion, which leads to airway mucus obstruction [1]. In the context of CF, a disease characterised by pathogenic variants of the cystic fibrosis transmembrane conductance regulator (CFTR) gene, mouse models fail to exhibit CF‐like lung pathology [2]. To address this gap, researchers developed a mouse model overexpressing the β‐subunit of the epithelial sodium channel (ENaC) under the wild‐type Clara cell‐specific promoter, known as β‐ENaC‐Tg mice [3]. These mice exhibit increased sodium absorption, producing airway surface dehydration. The dehydration subsequently reduces mucociliary clearance, leads to mucus blockages, the transformation of goblet cells, and persistent inflammation in the airways [4]. These mice serve as a model of muco‐obstructive lung disease, chronic bronchitis, and emphysema.

β‐ENaC‐Tg mice develop early onset and chronic airway inflammation, characterised by airway neutrophil and macrophage activation [1, 5, 6, 7]. They develop epithelial remodelling and mucus hypersecretion, evidenced by goblet cell metaplasia and elevated airway mucins in the first 5 weeks of age. Chronic airway inflammation is associated with emphysema‐like structural lung disease, including distal airspace enlargement and alveolar wall destruction [4, 5]. Lung function studies show airflow obstruction and increased compliance, with computed tomography (CT) images showing reduced density of lung parenchyma [1]. Surviving adult β‐ENaC‐Tg mice have also been demonstrated to develop chronic bronchitis and emphysema, exhibiting increased lung volumes, distal airspace enlargement, and increased lung compliance [4].

Preclinical lung function assessments using animal models play a crucial role in advancing our understanding of the underlying mechanisms of pulmonary disease. One commonly utilised tool for assessing lung function in animal models, specifically rodents and small animals, is the flexiVent system developed by SCIREQ. This system allows researchers to conduct various lung function assessments by controlling the animal's ventilation and applying different test perturbations [8]. Functional assessments, such as those made by the forced‐oscillation technique (FOT), can be delivered as either single frequency or broadband perturbations, and data fitted to the single compartment or constant phase models, respectively. The single compartment model provides assessments of total respiratory resistance and compliance, whilst the constant phase model offers a deeper understanding of airway and tissue mechanics, quantifying tissue damping, tissue elastance, and Newtonian resistance (central airway resistance) [9]. Moreover, the construction of pressure‐volume loops can be used to characterise lung distensibility across the inspiratory capacity, and the use of the negative‐pressure forced‐expiration extension allows measurement of forced vital capacity (FVC), forced expired volume (FEV) and forced expired flow (FEF) data. This flexiVent assessment provides information about the dynamic and static properties of the respiratory system in animal models, aiding in the identification of deviations from normal lung function.

While functional assessments can provide information about the mechanical properties of the respiratory system, imaging techniques, such as CT and magnetic resonance imaging (MRI), offer researchers the ability to both visualise and quantify structural changes [10, 11]. These imaging modalities provide visual insights into the lung architecture and pinpoint abnormalities such as mucus plugging, air trapping, bronchiectasis, and emphysema. Mathematical models of lung mechanics can provide a wealth of insights into the physical properties of the respiratory system. While some mathematical models can partition the lung's response into airway and tissue mechanics components [12], the spatial resolution of these models remains limited. Traditional imaging, while valuable, provides a static snapshot of the lung. Many lung diseases start locally, affecting lung parenchyma and small airways in a heterogeneous manner, with functional effects often masked by lung compensatory mechanisms until significant lung structure is compromised [8]. Recent advancements have led to the development of X‐ray velocimetry (XV), which allows researchers to directly observe and quantify the movement of lung tissue during a breath, providing real‐time functional ventilation data [13, 14].

β‐ENaC‐Tg mice were the first model used to demonstrate the translation of XV technology from a synchrotron facility [15] to a laboratory setting [16, 17]. In the initial studies, the authors demonstrated that measurements such as regional airflow and tissue expansion in vivo could be captured in live mice [16]. More recently, the development of the Permetium preclinical XV scanner (4DMedical, Australia), combined with custom XV analysis software, allows the motion of the lung tissue and therefore local ventilation to be measured in a laboratory setting. This technology reveals the complete dynamics of airflow across the lung during a single breath cycle [18, 19]. In the period since those proof‐of‐principle XV studies using β‐ENaC‐Tg mice were performed, a full lung function characterisation of this mouse model has not been completed.

The primary study aim was to conduct a comprehensive lung function analysis of adult β‐ENaC‐Tg mice, employing both XV assessments and flexiVents. We hypothesised that XV would quantify and visually represent the extent and location of ventilation alterations in β‐ENaC‐Tg mice compared to wild‐type mice. We expected these alterations to align with the parameterised lung mechanics obtained from flexiVent assessments.

## 2 Methods

### 2.1 Animals

All animal procedures were approved by the South Australian Health and Medical Research Institute (SAHMRI) animal ethics committee under application SAM‐23‐020 and were performed in accordance with ARRIVE guidelines [20]. β‐ENaC‐Tg mice (C3H × C57BL/6N background strain) and normal littermates (wild‐types) were provided by Charité‐Universitätsmedizin Berlin, Berlin, Germany [3]. Male and female β‐ENaC‐Tg (*n* = 14; 4 male and 10 female) and wild‐type (*n* = 13; 4 male and 9 female) mice 12–14 weeks of age were used. β‐ENaC‐Tg mice were identified by PCR [3]. All XV imaging and flexiVent lung function tests were performed at the SAHMRI Preclinical Imaging and Research Laboratories (PIRL, South Australia).

### 2.2 X‐Ray Velocimetry (XV) Imaging

XV imaging was performed as previously described [21, 22, 23]. Briefly, mice were anaesthetised with a mixture of 75 mg/kg of ketamine (Ceva, Australia) and 1 mg/kg medetomidine (Ilium, Australia), delivered by intraperitoneal (i.p) injection. Once anaesthetised, the mice were prepared by performing a tracheostomy followed by cannulation with a cut‐down endotracheal tube (ET; 18 Ga BD Insyte plastic cannula bevel‐cut to 15 mm length). The mice were then positioned upright (head‐high) in an animal holder and placed onto the rotation stage of the Permetium preclinical scanner (4DMedical, Australia). Mice were ventilated using a pressure‐controlled small animal ventilator (4DMedical Accuvent 200, Australia) set to a peak inspiratory pressure of 14 cmH_2_O, a positive end‐expiratory pressure of 2 cmH_2_O, and a breathing rate of 150 breaths/min (200 ms inspiration and 200 ms expiration, I:E ratio of 1:1).

The scanner used a Rigaku MM09 x‐ray source (40 kV, 2 mA) with a 40 μm molybdenum filter. The mice were 1680 mm upstream of the Varex 2020DX flat‐panel detector. Six thousand images were taken as the mice were continuously rotated 360° in the beam, with images taken at 10 phase points equally spaced across the breadth. A single XV acquisition took 4 min and 20 s.

XV analysis was performed using a three‐dimensional cross‐correlation based method that measures tissue displacement throughout the breath (4DMedical, Australia) using a proprietary algorithm based on previously published work [19, 24]. Specific ventilation (the percentage volume change of a region of lung compared to its size at the beginning of the breath) was calculated at around 6000 points across the lungs. The 4DMedical software produces a ventilation report containing slices through the lung that show 2D maps of local ventilation. The specific ventilation data is artificially coloured with average ventilation in green, below average in red, and above average ventilation in blue. In addition, a volume of specific ventilation data was also provided for each animal (see data repository for ventilation reports and 3D volumes). From this volume, global metrics such as mean specific ventilation (MSV) and ventilation heterogeneity (VH; the interquartile range divided by the mean, which gives a measure of the ventilation variance across the lung) were calculated. For XV, the ventilation defect percentage (VDP) describes the percentage of the lung with a specific ventilation below a fixed threshold. Typically, this threshold is 60% of the mean specific ventilation for that animal/patient [19, 21, 22, 24]. However, changes in mean specific ventilation between animals cause the VDP threshold to move arbitrarily for each animal, so here we normalise this to use 60% of the mean specific ventilation of the wild‐type group to define a fixed threshold and report the normalised ventilation defect percentage (nVDP) as first described by Reyne et al. (2024) [22].

To explore regional variations, these parameters were also calculated on subsets of the lung volume. A mask that contained only regions of the lungs at least three voxels away from the lung surface in the areas near the rib cage and from all voxels at the surfaces around the heart cavity was created. This was done by applying a closing filter of kernel size 9 × 9 × 9 to a binary lung mask, followed by an erosion filter with size 3 × 3 × 3. This mask was used to split the lungs into outer and inner regions, with the outer regions containing 61% of the lung by volume on average. The total lung volume at peak exhalation (including both lung tissue and air) was calculated from the size of the XV volumes produced.

### 2.3 flexiVent Respiratory Mechanics

Following the acquisition of XV scans, lung function assessments were conducted using a flexiVent FX small animal ventilator (SCIREQ, Montreal, Canada) with the mice in a supine position. The ventilator was equipped with an FX2 mouse module and negative pressure‐driven forced expiration (NPFE) extension and operated using flexiWare v8.0 software. Subjects were integrated into the flexiVent via the 18G cannula and ventilated at a rate of 150 breaths/min, an inspiratory ratio of 2:3, tidal volume set at 10 mL/kg, and a positive end expiratory pressure (PEEP) of 3 cmH_2_O. The lung mechanics evaluations were executed utilising SCIREQ's automated algorithms, which were configured within a mouse mechanics scan script that was repeated three times, as previously described [25]. Briefly, the script performed a deep inflation manoeuvre, followed by single frequency (SnapShot‐150) and broadband (Quick Prime‐3) oscillations and a pressure volume loop (PVs‐P). An NPFE perturbation was then executed to generate a flow‐volume loop and measure parameters such as forced expiratory volume (FEV), forced vital capacity (FVC), and peak expiratory flow (PEF). For each parameter, three measurements were made for each mouse, and these were averaged. Data was excluded if the coefficient of determination—a measure of model fit—was less than 0.9 for each model. After lung analysis, mice were humanely killed by i.p. overdose of lethabarb (150 mg/kg, Virbac, Australia).

### 2.4 Tissue Analysis

Lungs were inflation‐fixed in situ using a 10% formalin buffer at 18 cmH_2_O for 2 min; lungs were then removed and placed into formalin for at least 24 h and transferred to 70% ethanol until processing. Samples were embedded in paraffin wax, and 5 μm slices were mounted onto slides and stained with Alcian Blue/Periodic Acid Schiff for the detection of mucus. Images were captured using a Nikon Eclipse Ci‐L plus Microscope with a Nikon DS F*i*3 camera and NIS‐elements D software (version 5.42.03).

### 2.5 Statistics

All statistical analyses were performed in R version 4.3.1 [26]. In keeping with the development of modern bio‐statistical analyses that better reveal and describe the clinical relevance of findings—as opposed to providing only binary *p‐value* decisions about statistical significance—we express our statistical findings in terms of estimated marginal means and 95% confidence intervals returned from linear models fitted to the data. For every flexiVent and XV parameter, differences in means between genotypes (and 95% confidence intervals) were estimated with linear regression models using the “lm” function, adjusting for age and sex. Estimated marginal means (averaged over the covariate distribution) were also derived and plotted. *Post hoc* pairwise comparisons for the fitted model were carried out using the “emmeans” package [27]. Table S1 shows the estimated differences in mean between the genotypes and 95% confidence intervals, and the actual *p‐value* for each parameter.

## 3 Results

### 3.1 Animals

In this study, β‐ENaC‐Tg and normal littermate (wild‐type) control mice were compared. The age and weight of the β‐ENaC‐Tg mice were not significantly different from the wild‐type mice (Table 1).

**TABLE 1:** Age and weight of the mice.

Genotype	Average age (days)	SD days (days)	Average weight (g)	SD weight (g)
β‐ENaC‐Tg	90.79	3.83	23.74	2.78
Wild‐type control	91.62	3.79	24.22	3.93

### 3.2 Histology

**FIGURE 1:**
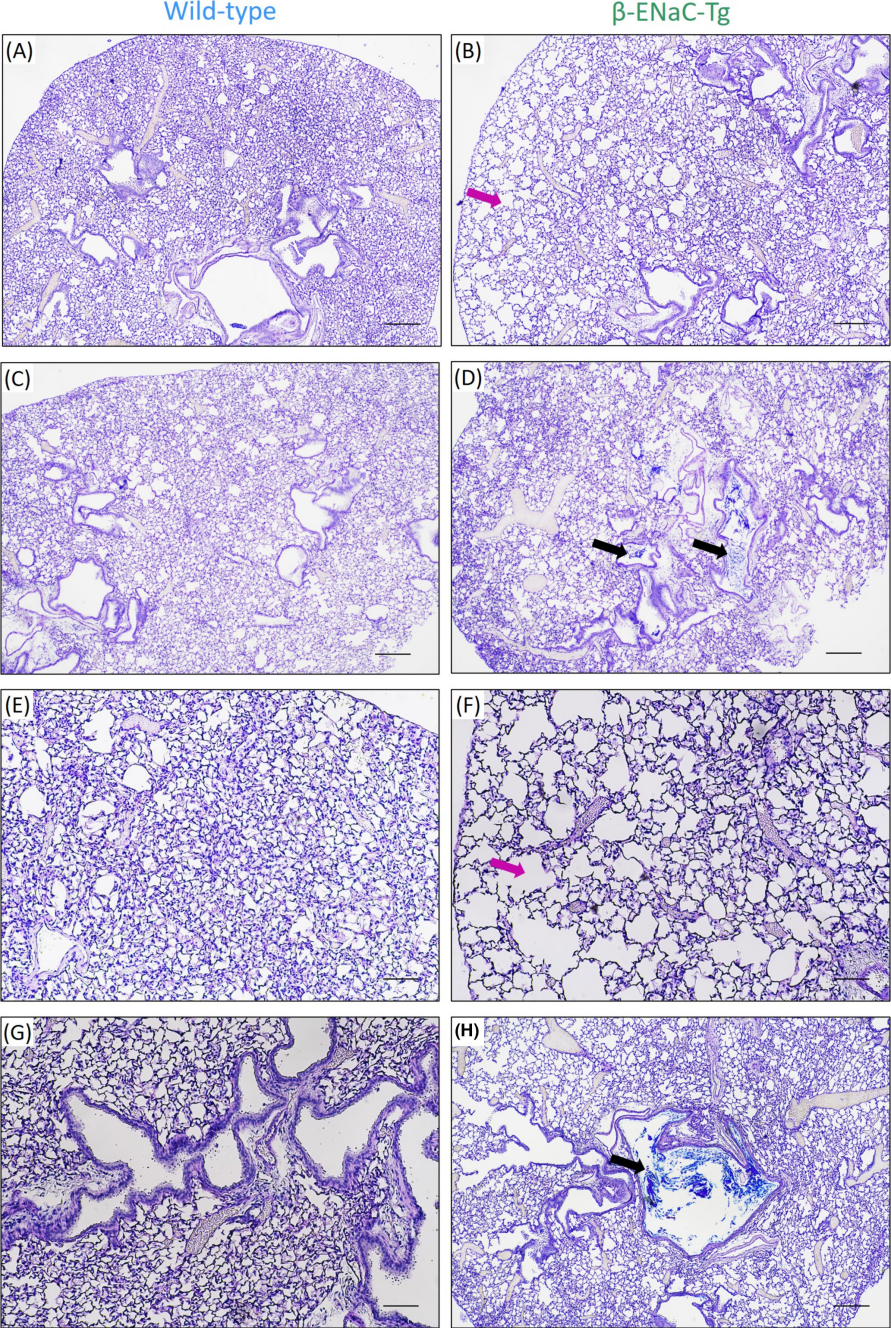
Alcian Blue/Periodic Acid Schiff staining of histological sections in four wild‐type and four β‐ENaC‐Tg mice. (A, C, E, G) wild‐type mice airways were normal, compared to (B, D, F, H) partially mucus‐obstructed airways (black arrows) and increased airspace (pink arrows) in β‐ENaC‐Tg mice. Scale bar A, B, C, D: 250 μm (40×), E, F, G, H: 100 μm (100×).

Airway luminal mucus was detected in all β‐ENaC‐Tg mice, primarily in the larger and middle airways, but no mucus was found in the littermate wild types (Figure 1). The mucus accumulation in the β‐ENaC‐Tg mice airways was heterogeneous, and when mucus was observed it did not fully obstruct or plug the airways. Evidence of emphysema, enlargement of airspace, was found in β‐ENaC‐Tg mice, with findings consistent with those previously reported [4].

### 3.3 X‐Ray Velocimetry (XV) Imaging

**FIGURE 2:**
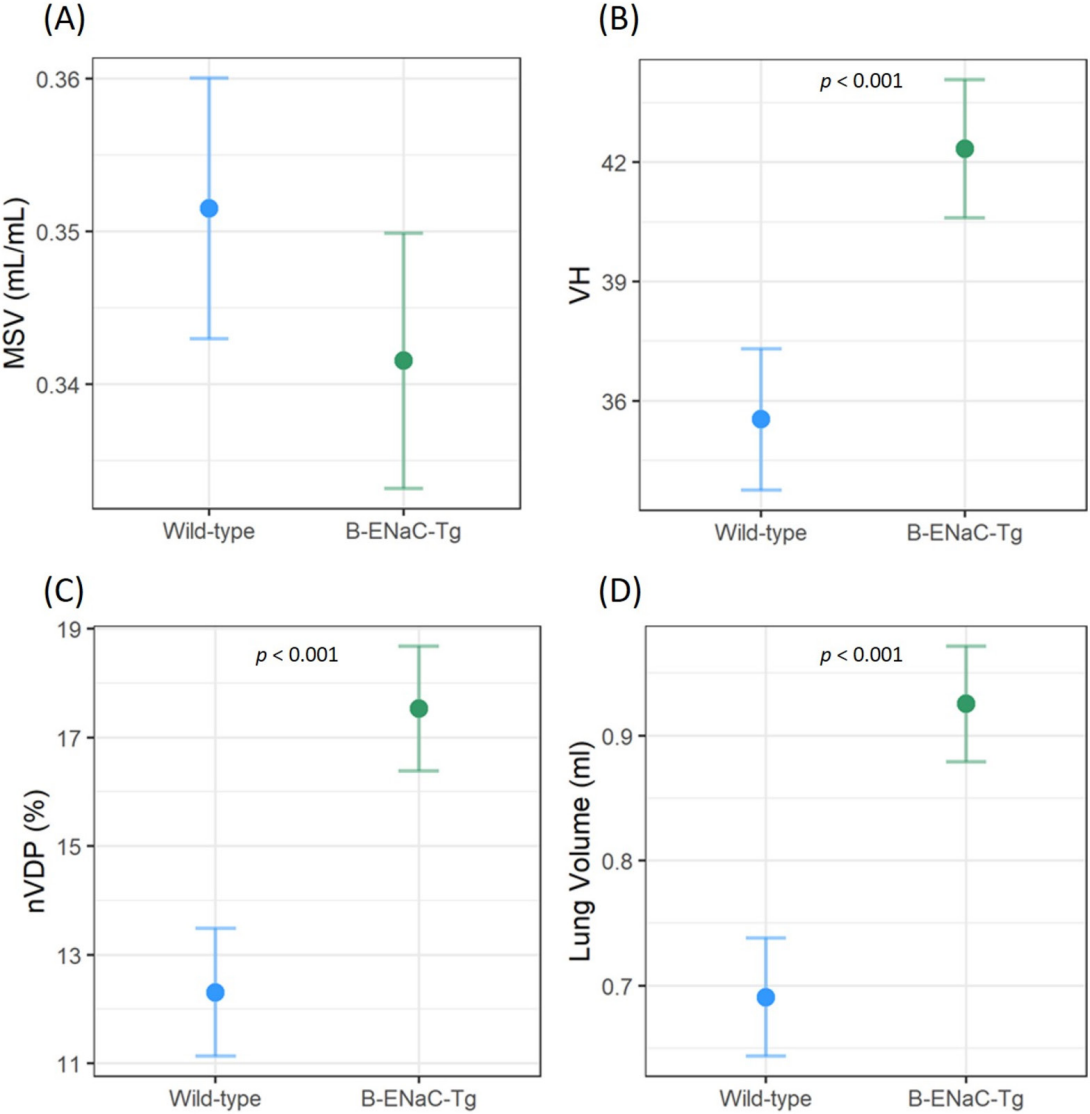
Whole lung XV measurements in wild‐type (blue) and β‐ENaC‐Tg (green) mice. (A) Mean specific ventilation (MSV), (B) Ventilation heterogeneity (VH), (C) normalised ventilation defect percentage (nVDP) and (D) the total lung volume (tissue and air) at end exhalation. (*n* = 13‐14/genotype, standard linear regression model, estimated means and 95% CIs).

XV analysis showed that the normalised ventilation defect percentage, ventilation heterogeneity, and lung volume were higher in the β‐ENaC‐Tg mice compared to the wild‐type mice, but there was no difference in mean specific ventilation (Figure 2).

**FIGURE 3:**
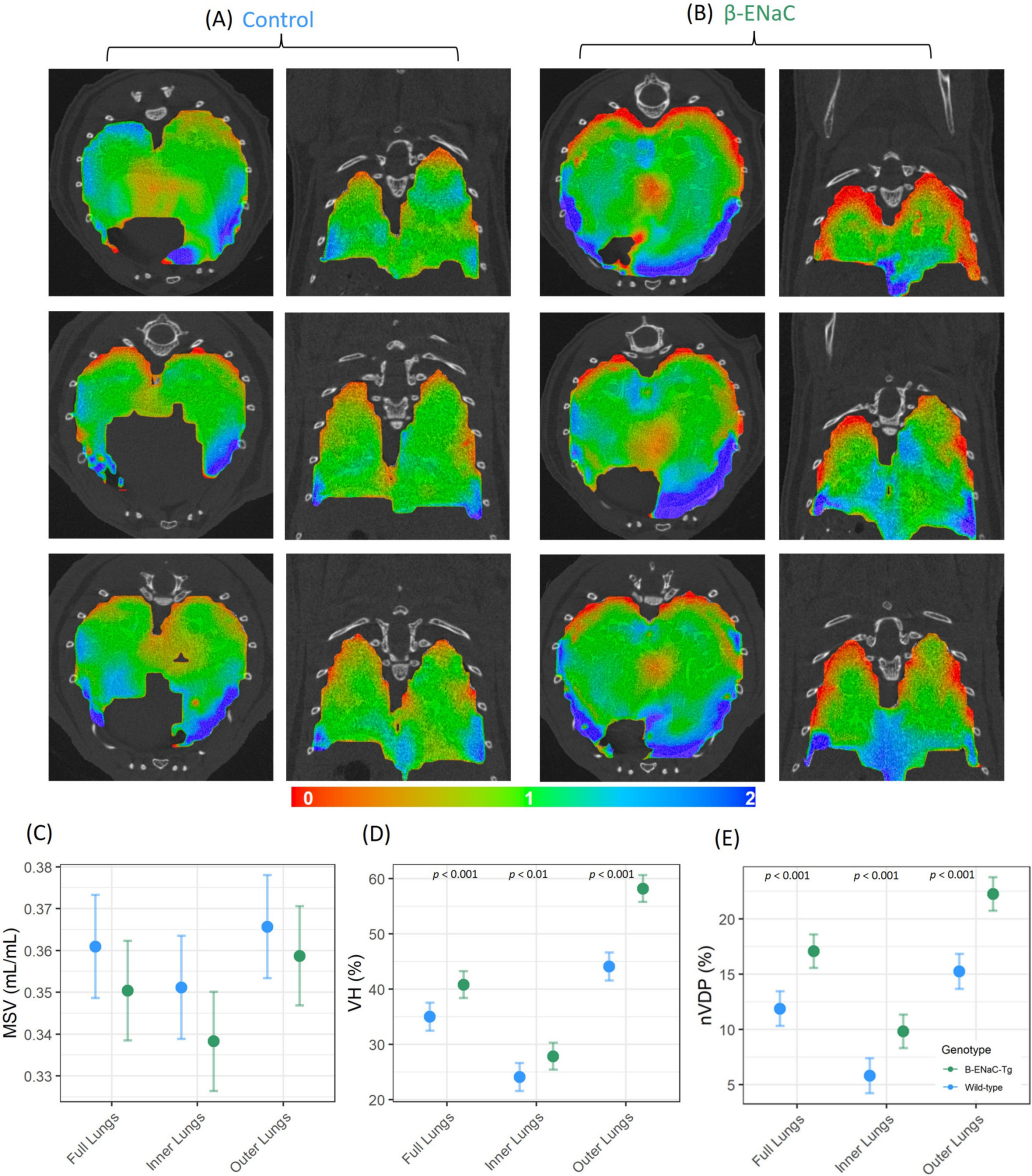
XV ventilation maps and metrics from wild‐type and β‐ENaC. The 2D ventilation slice (from similar location) mappings from 4DMedical ventilation reports, derived from 3D XV volume data, from representative (A) wild‐type and (B) β‐ENaC‐Tg mice in the transverse and coronal directions. Green indicates average ventilation, red below average ventilation and blue above average ventilation. Graphs show (C) Mean specific ventilation (MSV), (D) ventilation heterogeneity (VH), and (E) normalised ventilation defect percentage (nVDP) (*n* = 13‐14/genotype, standard linear regression model, estimated means and 95% CIs).

A visual examination of the XV ventilation maps indicated that the β‐ENaC‐Tg mice appeared to exhibit more areas of extreme high and low ventilation in the areas of the lung closest to the rib cage (Figure 3A,B). Based on this finding, the lungs were split into an inner core region (average 39% of total lung volume) and an outer region nearer to the rib cage. The outer and inner regions displayed obvious differences in the ventilation heterogeneity and normalised ventilation defect percentage between β‐ENaC‐Tg and wild‐type mice (Figure 3). PDF ventilation reports for all animals are provided in the data repository.

### 3.4 flexiVent Respiratory Mechanics

**FIGURE 4:**
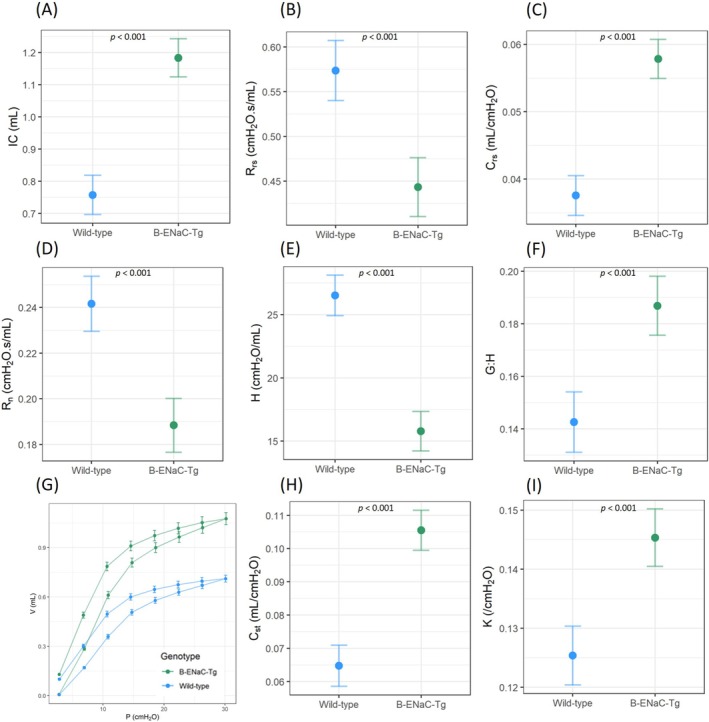
FlexiVent assessments of the respiratory system in wild‐type and β‐ENaC‐Tg mice. Deep inflation was used to produce the (A) inspiratory capacity (IC). The single frequency forced oscillation measured the (B) total respiratory system resistance (R_rs_) and (C) total respiratory system compliance (C_rs_). The broadband forced oscillation measured the (D) Newtonian resistance (R_n_), (E) tissue elastance (H) and (F) tissue hysterisivity (G:H) of the peripheral lung compartment. The pressure volume loop (G) was used to determine the static compliance (H) and K‐parameter (I) (from fitting the Salazar Knowles equation to the deflation limb of the PV loop). (*n* = 13–14/genotype, standard linear regression model, estimated means and 95% CIs).

The deep inflation manoeuvre showed that the β‐ENaC‐Tg mice had a higher inspiratory capacity compared to wild‐types (Figure 4A), and the single frequency forced oscillation (single compartment model) demonstrated higher total respiratory system compliance (C_rs_) and lower total respiratory system resistance (R_rs_) in β‐ENaC‐Tg mice compared to wild‐type (Figure 4B,C). The broadband forced oscillation (constant phase model) demonstrated that Newtonian (central airway) resistance (R_n_), tissue elastance (H), tissue damping (G), and tissue hysterisivity (G/H) were significantly different in β‐ENaC‐Tg mice compared to wild‐type mice (Figure 4D–F). Average pressure volume loops constructed from mean data showed an upward shift in the pressure‐volume relationship of β‐ENaC‐Tg mice, which was different from wild‐type mice (Figure 4G–I). The static compliance (C_st_) and shape parameter K (shift in PV loop) were higher in the β‐ENaC‐Tg mice.

**FIGURE 5:**
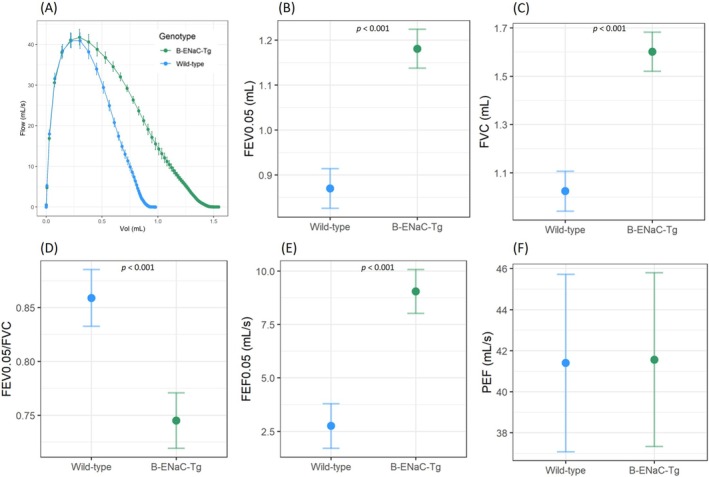
Negative pressure‐driven forced expiration manoeuvres showed differences in β‐ENaC‐Tg mice compared to wild types. (A) Representative mean flow‐volume curves from wild‐type and β‐ENaC‐Tg mice. (B) Forced expiratory volume (0.05 s), (C) FVC, (D) FEV_0.05_/FVC, (E) forced expiratory flow (0.05 s), and (F) peak expiratory flow (*n* = 13‐14/genotype, standard linear regression model, estimated means and 95% CIs).

When the negative pressure forced expiratory test was applied, the forced expiratory volume at 0.05 s (FEV_0.05_), forced vital capacity (FVC), FEV_0.05_/FVC, and forced expiratory flow at 0.05 s (FEF_0.05_) were higher in β‐ENaC‐Tg mice (Figure 5). The peak expiratory flow (PEF) however, was not significantly different between β‐ENaC‐Tg mice and wild types.

## 4 Discussion

Previous studies with β‐ENaC‐Tg mice have provided partial data on flexiVent‐derived parameters [4, 15, 28, 29, 30, 31, 32, 33, 34]. Here, we comprehensively describe findings from a wide range of flexiVent manoeuvres for a thorough characterisation of lung function. In addition, we utilise XV to measure ventilation heterogeneity differences and to highlight the advantages of visually representing these changes in lung function.

Forced‐oscillation assessments of the β‐ENaC‐Tg mice—including single frequency (single compartment model) and broadband (constant phase model) methods—demonstrated mechanical properties typical of an emphysematous model: a highly compliant lung with increased lung volumes. Our results showed that β‐ENaC‐Tg mice have a higher inspiratory capacity (from deep inflation), along with a higher total respiratory system compliance and decreased total respiratory system resistance as assessed by the single compartment model. These parameters reflect the contributions of the lung tissue, chest wall, and airways. The broadband forced oscillation (constant phase model) provided further insights by distinguishing between lung mechanics in central conducting airways and alveolar tissues. Newtonian resistance, which primarily represents the resistance of the conducting airways, was lower in the β‐ENaC‐Tg mice, suggesting they have larger diameter airways than the wild‐type animals. Previous studies have shown that adult β‐ENaC‐Tg mice have enlarged distal airspaces compared to wild‐type mice [4]. Lower tissue damping (G) and elasticity (H) in β‐ENaC‐Tg mice compared to wild types indicated a loss of structural integrity in the peripheral airways and lung parenchyma. Additionally, a higher tissue hysteresivity (G:H) in β‐ENaC‐Tg mice compared to wild types indicates lung heterogeneity, suggesting shifts in ventilation patterns across different lung regions. This finding aligns with the ventilation heterogeneity observed in the periphery of the lung in β‐ENaC‐Tg mice using XV imaging.

To further assess the compliance of the respiratory system, pressure‐driven pressure volume loops were created by inflating the lung in a stepwise manner, from PEEP (3 cmH_2_O) to 30 cmH_2_O. The β‐ENaC‐Tg mice displayed an upward shift in the pressure‐volume relationship, higher static compliance, and K‐parameter values than wild‐type mice [4, 31, 33]. Static compliance reflects the intrinsic distensibility of the respiratory system across the entire inspiratory capacity, and the K‐parameter describes the curvature of the upper portion of the deflation limb of the PV loop (another representation of compliance). These results are consistent with the forced oscillation technique results, describing β‐ENaC‐Tg mice as having significantly more compliant lungs.

The NPFE manoeuvre generated a higher maximal inflation volume in the β‐ENaC‐Tg mice than in the wild‐type mice, which matched the inspiratory capacity assessments. FEV0.05, FVC, and FEF were higher in the β‐ENaC‐Tg mice compared to the wild‐type mice. Although this was consistent with previous findings [28, 30, 32], it is unusual for a high FEV to be observed in CF or emphysema patients [35]. Here it is likely that the increased compliance increases FVC (due to the larger inspiratory capacity) and thus FEV0.05, which is also consistent with the reduced total respiratory resistance reported by the single compartment model. Interestingly, despite histological evidence of increased mucus accumulation in the β‐ENaC‐Tg mice, the lung function testing indicated reduced resistance and no evidence of obstruction during the NPFE manoeuvre. This is unsurprising, as it is well established that forced expiratory manoeuvres are particularly poor at measuring peripheral airway function [36, 37].

A key difference between clinical spirometry data and data derived from the NPFE extension is the manner in which the data is acquired. In clinical spirometry, patients consciously and voluntarily perform exhalations, whereas NPFE assessments are made on a passive ventilated subject. Here the NPFE generates data by first inflating the lungs to 30 cmH_2_O (inspiratory capacity) and then rapidly withdrawing the air from the subject via application of −55 cmH_2_O of negative pressure to the endotracheal tube. This negative pressure application can potentially induce dynamic airway collapse and restrict airflow in a manner that cannot occur during conscious exhalation in clinical settings. Subjects with a large loss of structural tissue integrity (high compliance, that is, emphysematous models) may be particularly prone to this effect [38]. Despite the increased compliance, there was no evidence of any expiratory flow limitation in the β‐ENaC‐Tg mice, which suggests that the physiological mechanisms that result in reduced compliance are different from those typically observed in emphysema and should be taken into consideration when using this mouse as a model of emphysema.

A longitudinal study by Mall (2008) from newborn to 6 weeks old revealed that in younger β‐ENaC‐Tg mice, lung volumes, alveolar architecture, and alveolar size remained relatively normal, despite mucus plugging [4]. However, in that study, as the mice aged, there was a subsequent increase in lung volume and distal airspace, ultimately leading to the development of emphysema. The development of emphysema is thought to be caused by several factors, including proteolytic damage of alveolar septi by proteases released from neutrophils and macrophages in chronic airway inflammation, such as neutrophil elastase (NE) and macrophage elastase (MMP12), and potentially other processes related to alveolar septation during development and the persistent hyperinflation of the lungs causing air trapping [4]. The mice in the present study were older, averaging 13 weeks old, and were therefore expected to have more severe emphysema than what was reported in Mall et al. (2008) [4]. Mucus plugging/accumulation, observed in previous studies with β‐ENaC‐Tg mice [3, 4], was also observed in this study. Data here support the association found in older people with CF, who can develop emphysema with advanced lung disease.

The normalised ventilation defect percentage was higher in β‐ENaC‐Tg mice than in the wild‐type animal, and the ventilation heterogeneity was also higher, signifying uneven or patchy airflow across the lungs caused by the CF‐like muco‐obstructive lung disease and emphysema phenotypes. Our findings align with the initial studies using β‐ENaC‐Tg mice to validate in vivo XV, where decreases in regional lung expansion were observed [15, 16, 17]. Further spatial analysis of the XV ventilation maps indicated that the outer regions of the lung were more heterogeneous and exhibited a higher normalised VDP compared to the rest of the lung. This could be due to the fact that the alveolar ducts located at the edges of the lungs in β‐ENaC‐Tg mice are wider than those in wild‐type, as found in the Blaskovic et al. (2023) study using micro‐CT [39], or due to the mucus plugging observed in the small airways. The difference in MSV values between the inner and outer regions was more pronounced in β‐ENaC‐Tg mice than in wild‐type mice, strengthening the argument for increased heterogeneity across the whole lung. Similarly, a study on emphysema in people with CF found that CF‐emphysema predominantly occurred in the subpleural regions [35], the areas of the lung closest to the ribcage.

XV analysis provided spatial information to identify the origins of these lung function changes in adult β‐ENaC‐Tg mice. In future studies, XV could be employed to track the changing lung phenotype observed in β‐ENaC‐Tg mice over time, quantifying the transition from the early mucus obstructive phenotype to the later emphysematous phenotype [4]. Such studies could provide valuable data for subsequent validation of XV imaging as a tool for monitoring lung disease in children with CF and other muco‐obstructive lung diseases. Further, investigating the effectiveness of mucolytics on the excess mucus present in β‐ENaC‐Tg mice on lung ventilation changes could offer insights into how ventilation changes in the lung after successful treatment [40]. Finally, advanced imaging processing approaches should also be developed to better interpret the spatial variations in the ventilation data produced by XV imaging.

In conclusion, the objective of this study was to extend the characterisation of lung function and mechanics in adult β‐ENaC‐Tg mice and pinpoint the location and extent of airway ventilation. Here we provide a comprehensive analysis of lung function in β‐ENaC‐Tg mice using flexiVent and XV technology. Histology demonstrated mucus accumulation in the airways and enlarged air spaces that were consistent with what has previously been reported [3]; the flexiVent showed increased compliance and reduced respiratory resistance, and XV demonstrated increased VH and VDP. XV functional lung imaging showed that β‐ENaC‐Tg mice have regions of altered ventilation in the peripheral regions of the lungs.

## Author Contributions

**Nicole Reyne:** conceptualization (equal), data curation (equal), formal analysis (equal), investigation (equal), methodology (equal), writing – original draft (lead). **Ronan Smith:** data curation (equal), formal analysis (equal), investigation (equal), software (equal), visualization (equal), writing – original draft (equal). **Patricia Cmielewski:** data curation (equal), formal analysis (equal), investigation (equal), writing – original draft (equal). **Nina Eikelis:** formal analysis (equal), writing – review and editing (equal). **Kris Nilsen:** formal analysis (equal), writing – review and editing (equal). **Jennie Louise:** formal analysis (equal), writing – review and editing (equal). **Julia Duerr:** resources (equal), writing – review and editing (equal). **Marcus A. Mall:** conceptualization (equal), funding acquisition (equal), resources (equal), writing – review and editing (equal). **Mark Lawrence:** formal analysis (equal), writing – review and editing (equal). **David Parsons:** conceptualization (equal), funding acquisition (equal), resources (equal), writing – review and editing (equal). **Martin Donnelley:** conceptualization (equal), data curation (equal), formal analysis (equal), funding acquisition (equal), investigation (equal), methodology (equal), supervision (equal), writing – original draft (equal).

## Ethics Statement

All animal procedures were approved by the South Australian Health and Medical Research Institute (SAHMRI) animal ethics committee under application SAM‐23‐020 and were performed in accordance with ARRIVE guidelines.

## Conflicts of Interest

M.D. and D.P. were involved in the research, development, and validation of the XV technology and have personally purchased shares in 4DMedical. N.E. and K.N. employed by 4DMedical. M.L. is employed by SCIREQ Scientific Respiratory Equipment Inc. M.A.M. has a patent on the β‐ENaC‐Tg mouse as an animal model for chronic obstructive pulmonary disease and cystic fibrosis.

## Supporting information


**Table S1.** Estimated difference of XV and flexiVent measurements in wild‐type and βENaC mice. Data are presented as estimated marginal mean and 95% confidence intervals, and the actual *p* value for each parameter. Positive values indicate the parameter was higher in βENaC mice than wild‐type.

**Visual Abstract** X‐ray velocimetry identifies peripheral ventilationchanges in β‐Epithelial‐Na‐Channel‐Tg mice

## Data Availability

The data are hosted in a repository at DOI: 10.25909/25814851.
