# Supplementary material for: Functional Lung Imaging Identifies Peripheral Ventilation Changes in ꞵ‐ENaC Mice
Source: Respirology. 2025 Feb 25;30(4):335–45. doi: 10.1111/resp.70009 (PMC11965024; doi:10.1111/resp.70009)
Supplement: Supplementary file 1 — Table S1. Estimated difference of XV and flexiVent measurements in wild‐type and βENaC mice. Data are presented as estimated marginal mean and 95% confidence intervals, and the actual p value for each parameter. Positive values indicate the parameter was higher in βENaC mice than wild‐type. [file RESP-30-335-s001.docx]

| βENaC - Wild-type | | | | | | |
| --- | --- | --- | --- | --- | --- | --- |
|  |  |  | estimate | 95% CI | | *p* value |
| XV | MSV | mL/mL | -0.01 | -0.02 | 0.00 | 0.08 |
|  | TV | mL | 0.07 | 0.05 | 0.10 | **<0.001** |
|  | VH | % | 6.80 | 4.42 | 9.18 | **<0.001** |
|  | VDP | % | 4.15 | 2.93 | 5.37 | **<0.001** |
| NPFE | FEV0.05 | mL | 0.31 | 0.25 | 0.37 | **<0.001** |
|  | FVC | mL | 0.58 | 0.47 | 0.69 | **<0.001** |
|  | FEV0.05/FVC |  | -0.11 | -0.15 | -0.08 | **<0.001** |
|  | FEF0.05 | mL/s | 6.29 | 4.89 | 7.69 | **<0.001** |
|  | PEF | mL/s | 0.16 | -5.62 | 5.94 | 0.95 |
|  | TPEF | s | 0.00 | 0.00 | 0.00 | 0.15 |
| Deep Inflation | IC | mL/100g | 0.43 | 0.34 | 0.51 | **0** |
| SnapShot-150 | Rrs | cmH2O.s/mL | -0.13 | -0.18 | -0.09 | **<0.001** |
|  | Crs | mL/cmH2O | 0.02 | 0.02 | 0.02 | **<0.001** |
|  | Ers | cmH2O/mL | -10.29 | -12.49 | -8.09 | **<0.001** |
| Quick-Prime 3 | Rn | cmH2O.s/mL | -0.05 | -0.07 | -0.04 | **<0.001** |
|  | G | cmH2O/mL | -0.76 | -1.07 | -0.44 | **<0.001** |
|  | H | cmH2O/mL | -10.72 | -12.86 | -8.59 | **<0.001** |
|  | Eta |  | 0.04 | 0.03 | 0.06 | **<0.001** |
| PVs-P | Cst | mL/cmH2O | 0.04 | 0.03 | 0.05 | **<0.001** |
|  | A | mL | 0.35 | 0.28 | 0.42 | **<0.001** |
|  | K | /cmH2O | 0.02 | 0.01 | 0.03 | **<0.001** |
|  | Area | mL/.cmH2O | 0.68 | 0.46 | 0.89 | **<0.001** |

**Supplemental files**

**Table 1: Estimated difference of XV and flexiVent measurements in wild-type and βENaC mice.** Data is presented as estimated marginal mean and 95% confidence intervals, and the actual *p* value for each parameter. Positive values indicate the parameter was higher in βENaC mice than wild-type.
