# Supplementary material for: Functional Lung Imaging Identifies Peripheral Ventilation Changes in ꞵ‐ENaC Mice
Source: Respirology. 2025 Feb 25;30(4):335–45. doi: 10.1111/resp.70009 (PMC11965024; doi:10.1111/resp.70009)
Supplement: Supplementary file 2 — Visual Abstract X‐ray velocimetry identifies peripheral ventilationchanges in β‐Epithelial‐Na‐Channel‐Tg mice [file RESP-30-335-s002.pdf]

X-ray velocimetry identifies peripheral ventilation changes in β-Epithelial-Na-Channel-Tg mice

Aim: Undertake a comprehensive lung function and mechanics analysis of adult β-ENaC mice, a model of muco-obstructive lung disease.

Methods: X-ray Velocimetry scans were acquired with a 4DMedical Permetium scanner, and lung mechanics were assessed with a Scireq flexiVent

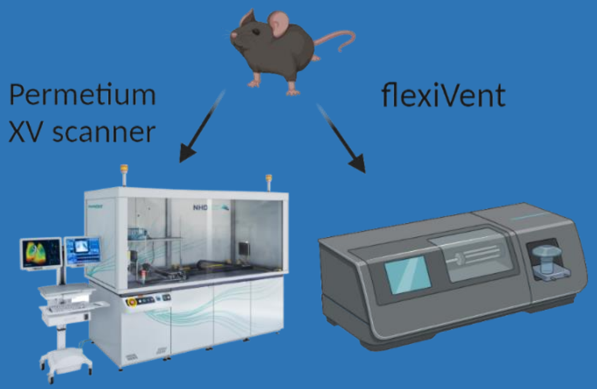

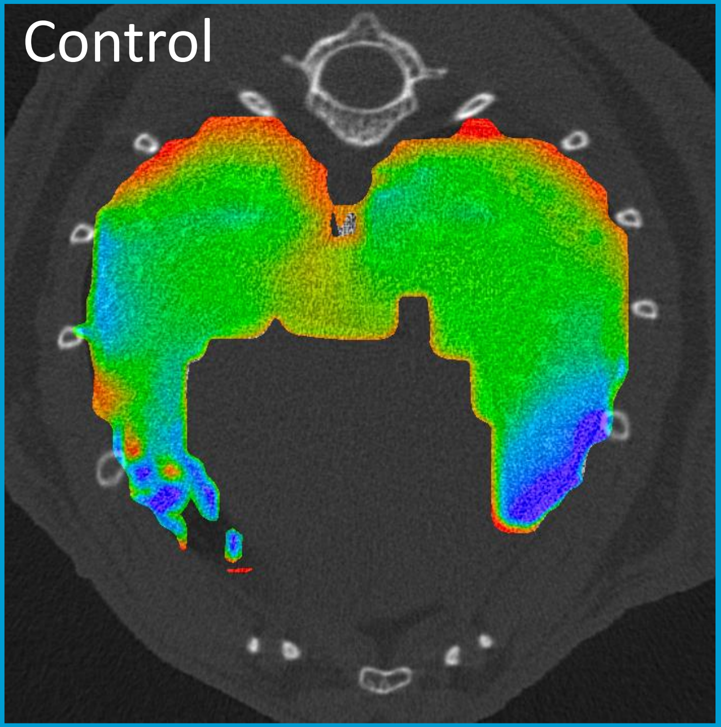

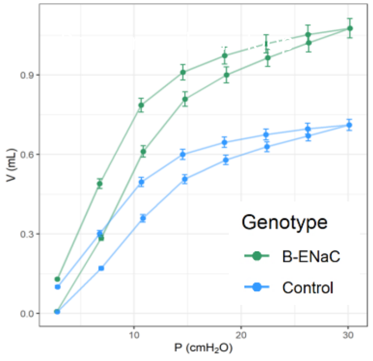

Flow volume curve

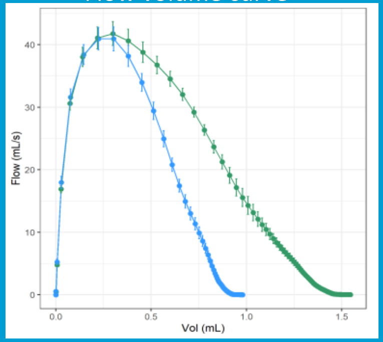

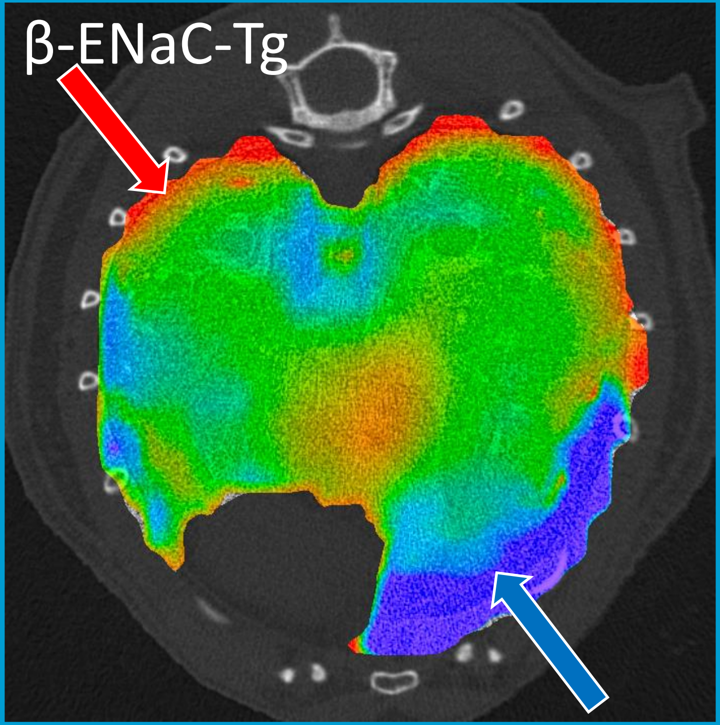

Results: β-ENaC-Tg mice exhibited patchy airflow across the lungs and regions of altered ventilation in the peripheral regions of the lungs. β-ENaC-Tg mice demonstrated mechanical properties of an emphysematous model.

Respirology

Functional lung imaging identifies peripheral ventilation changes in β-ENaC mice. Reyne et al., Respirology; 2025, DOI: 10.1111/resp.70009
